# Association Between Statin Use and Progression of Arterial Stiffness Among Adults With High Atherosclerotic Risk

**DOI:** 10.1001/jamanetworkopen.2022.18323

**Published:** 2022-06-17

**Authors:** Yan-Feng Zhou, Yanxiu Wang, Guodong Wang, Zhen Zhou, Shuohua Chen, Tingting Geng, Yan-Bo Zhang, Yi Wang, Jun-Xiang Chen, An Pan, Shouling Wu

**Affiliations:** 1 Department of Epidemiology and Biostatistics, School of Public Health, Tongji Medical College, Huazhong University of Science and Technology, Wuhan, China; 2 Department of Cardiology, Kailuan General Hospital, North China University of Science and Technology, Tangshan, China; 3 Menzies Institute for Medical Research, University of Tasmania, Hobart, Australia

## Abstract

**Importance:**

Limited studies have investigated the association between statin use and progression of arterial stiffness, a key player in the pathophysiology of cardiovascular disease.

**Objective:**

To examine the association between statin use and progression of arterial stiffness in Chinese adults with high atherosclerotic risk measured by brachial-ankle pulse wave velocity (baPWV).

**Design, Setting, and Participants:**

This retrospective cohort study enrolled 5105 adults with high atherosclerotic risk from the Kailuan General Hospital from 2010 to 2020. Data were analyzed from February 2021 to April 2022.

**Exposures:**

Statin use information was retrieved from electronic medical records from 2010 to 2020, and statin users were those who have been prescribed any statin medications at least 6 months before baPWV measurements. Statin users were 1:1 matched with non–statin users by propensity score method.

**Main Outcomes and Measures:**

Progression of baPWV was assessed using the absolute difference between baseline and follow-up baPWV, divided by the follow-up time in years. Multivariable linear regression models were used to estimate the association between statin use and arterial stiffness.

**Results:**

Among 5105 adults with assessment of baPWV (mean [SD] age: 60.8 [9.7] years; 3842 [75.3%] men and 1263 [24.7%] women), 1310 statin users were matched with 1310 non–statin users (mean [SD] age, 63.2 [9.3] years). Compared with non–statin users, statin users were associated with significantly lower baPWV at baseline (difference: −33.6 cm/s; 95% CI, −62.1 to −5.1 cm/s). Among 1502 adults with repeated assessment of baPWV, 410 statin users were matched with 410 non–statin users (mean [SD] age, 62.9 [9.2] years). Compared with non–statin users, statin users had significantly slower progression of baPWV (difference, −23.3 cm/s per year; 95% CI, −40.6 to −6.0 cm/s per year) during a mean (SD) follow-up of 4.8 (2.7) years. A significantly slower progression of baPWV was observed in continuous statin users (difference, −24.2 cm/s per year; 95% CI, −42.2 to −6.3 cm/s per year) and high adherent users (difference, −39.7 cm/s per year; 95% CI, −66.9 to −12.4 cm/s per year), but not in discontinued users (difference, −17.3 cm/s per year; 95% CI, −52.4 to 17.8 cm/s per year) and low adherent users (difference, −17.9 cm/s per year; 95% CI, −36.5 to 0.7 cm/s per year), compared with non–statin users.

**Conclusions and Relevance:**

In this cohort study, statin use was associated with slower progression of arterial stiffness in Chinese adults with high atherosclerotic risk.

## Key Points

**Question:**

Is statin use associated with progression of arterial stiffness as measured by brachial-ankle pulse wave velocity (baPWV)?

**Findings:**

In this cohort study assessing baPWV of 5105 adults with high atherosclerotic risk, the propensity-score matching yielded 1310 pairs of statin users and non–statin users for the analysis of baseline baPWV and 410 pairs for the analysis of progression of baPWV, respectively. The study found that statin use was associated with lower baseline baPWV (−33.6 cm/s) and slower progression of baPWV (−23.3 cm/s per year), compared with non–statin users.

**Meaning:**

These findings suggest that statin use was associated with slower progression of arterial stiffness.

## Teaser

This cohort study examines the association between statin use and the progression of arterial stiffness in 5105 Chinese adults with high atherosclerotic risk.

## Introduction

Arterial stiffness, an early detectable manifestation of adverse structural and functional changes in the vessel wall, is an independent predictor of overall cardiovascular risk and mortality.^1^ Brachial-ankle pulse wave velocity (baPWV), a valid, noninvasive clinical measure for arterial stiffness, has been increasingly used in epidemiological studies and is shown to be significantly associated with cardiovascular disease (CVD) and all-cause mortality.^2^

Atherosclerotic risk factors, predominantly dyslipidemia, type 2 diabetes, high blood pressure, obesity, and a cluster of these conditions (metabolic syndrome), are the main contributors to an accelerated progression of arterial stiffness.^3,4,5^ Statins, the first-line lipid-lowering therapies used widely to prevent CVD,^6,7^ serve as a promising pharmacological strategy to improve arterial stiffness. However, recent reviews reported inconsistent findings regarding the association between statin use and arterial stiffness, with some studies^8,9^ showing positive effects and others reporting null effects. The robustness of the evidence was limited by relatively small sample sizes (generally <100)^10,11,12,13,14,15,16,17,18,19,20^ and short-term follow-up durations (generally ≤6 months).^10,11,12,19,20^ In addition, no study has evaluated the association between statin use and progression of arterial stiffness.

In the present study, we used electronic medical records (EMRs) from the Kailuan General Hospital in a large community-based cohort in China to determine associations of statin use with arterial stiffness and its progression, measured by baPWV,^1^ in adults with high atherosclerotic risk.

## Methods

The Kailuan study (trial registration number ChiCTR-TNRC-11001489) was approved by the ethics committee of the Kailuan General Hospital. All participants signed a written informed consent form. This cohort study complies with the Declaration of Helsinki^21^ and follows the Strengthening the Reporting of Observational Studies in Epidemiology (STROBE) reporting guideline.

### Study Participants and Design

This study was embedded in the Kailuan study, a large, dynamic, community-based cohort study in Tangshan, China. The study design and procedures were detailed previously.^22^ Briefly, participants from the Kailuan Group received health checkups and questionnaires in the Kailuan General Hospital and 10 affiliated hospitals and clinics between 2006 and 2007 and were followed up with every 2 years. All employees and retirees in the Kailuan Group were obliged to enroll in the Urban Employee Basic Medical Insurance, and drug treatment could be partly reimbursed by health insurance.^23^

For the present study, we included participants who received health checkups and questionnaires in the Kailuan General Hospital, the largest and only tertiary care hospital in the Kailuan Group. Since the 2010 to 2011 cycle, participants at higher risks of peripheral arterial disease, meaning those with at least 1 risk factor—hypertension, diabetes, dyslipidemia, obesity, or metabolic syndrome—were invited to measure vascular health with baPWV measurements (eMethods in the Supplement).^24^ The date of the first baPWV measurement from 2010 to 2020 was considered as the baseline. Participants who had undertaken baseline baPWV measurements were invited to take follow-up measurements of baPWV.^25^

**Figure 1.**
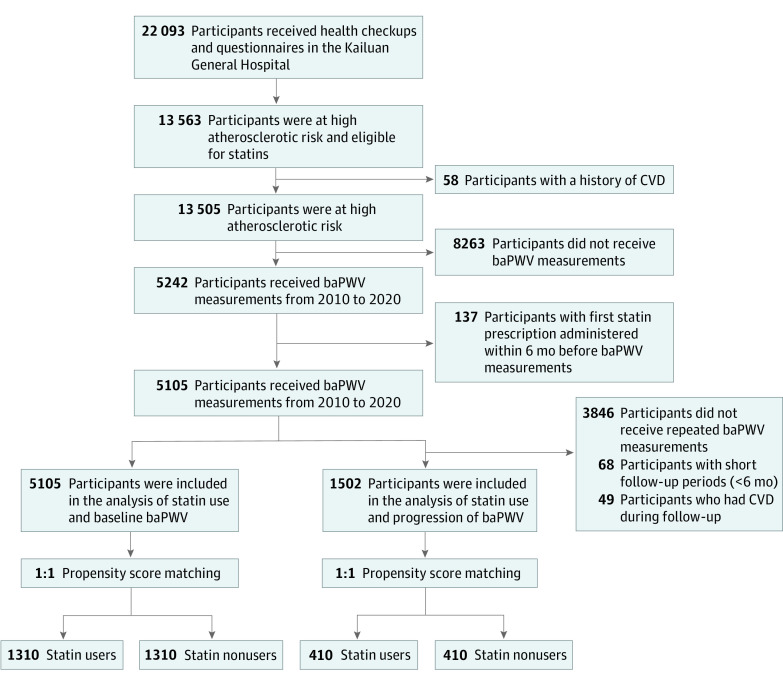
Participant Flowchart Abbreviations: baPWV, brachial-ankle pulse wave velocity; CVD, cardiovascular disease.

Initially, 22 093 participants received health checkups and questionnaires in the Kailuan General Hospital. Among them, 13 563 adults were eligible for the study. After excluding participants with a history of CVD who were identified via self-reports and EMRs (n = 58), those who did not receive baPWV measurements (n = 8263) and had their first statin prescription administered within 6 months before baPWV measurements (n = 137), 5105 participants were included in the analysis of statin use and baseline baPWV. In the analysis of statin use and progression of baPWV, we further excluded 3486 participants who did not undertake repeated assessments of baPWV, 68 participants with short follow-up periods (<6 months), and 49 participants who had CVD during follow-up, leaving 1502 participants in the final analytic cohort. The flowchart is shown in Figure 1.

### Statin Use

According to clinical guidelines,^26,27,28^ statin therapy was recommended in addition to lifestyle therapy for patients with hypertension, type 2 diabetes, dyslipidemia, obesity, or metabolic syndrome. Linking to the EMRs from January 1, 2010, to December 31, 2020, we identified the use of statins, antidiabetic, and antihypertensive drugs, which were identified according to their generic or trade name; drug names were adjudicated by the study team.

In our study, statin users were those who have been prescribed any statin medications at least 6 months before baPWV measurements.^29^ To explore associations of different statin groups with baPWV, statin users were subcategorized based on statin discontinuation, statin adherence, and statin type. Statin discontinuation was identified as the absence of statin prescriptions for 12 consecutive months within the first 2 years of outcome measurements (ie, baPWV measurements).^30^ Statin adherence was measured by medication possession ratio (MPR). For dichotomous analyses, high adherence was defined as an MPR of 80% or greater as per previous literature.^29,31^ Given a putative role of statin lipophilicity on cardiovascular outcomes, we also performed comparisons between hydrophilic statin users (ie, pravastatin and rosuvastatin) and lipophilic statin users (ie, atorvastatin, simvastatin, fluvastatin, lovastatin, and pitavastatin).^32^ In addition, statin users were further stratified based on the achieved low-density lipoprotein cholesterol (LDL-C) goal (<131.3 mg/dL and ≥131.3 mg/dL; to convert to mmol/L, multiply by 0.0259)^27^ during follow-up in the analysis of statin use and progression of arterial stiffness.

### baPWV Measurement

baPWV was measured at baseline and repeatedly during the follow-up. A BP-203 RPE III networked arterial stiffness detection device was used for baPWV measurement following the recommended standard procedures, as detailed elsewhere.^25^ In brief, the measurement was undertaken by trained nurses between 7 am and 9 am on the examination day, following the standard operation procedures. After not smoking and sitting for at least 5 minutes in a room with the temperature controlled between 22 °C and 25 °C, participants wore thin clothes and were asked to lie down on the examination couch in a supine position and be quiet during the measurement. Four cuffs were wrapped around the upper arms and ankles and connected to a plethysmographic sensor (volume pulse form) and oscillometric pressure sensor. The maximum of left and right sides of baPWV was used for analysis, and the methods for baPWV measurement remained the same for all participants. Progression of baPWV was assessed using the absolute difference between values of the baPWV at baseline and during the follow-up period, divided by the follow-up time in years.^33^

### Assessment of Covariates

Covariates were collected via self-reported questionnaires (ie, age, sex, educational level, smoking and drinking status, physical activity, salt intake, and medical comorbidities), basic anthropometric measurements (ie, body height and weight, heart rate, and mean arterial pressure [MAP]), and blood tests (ie, plasma glucose level, lipids level, high-sensitivity C-reactive protein [hs-CRP] level) during the biennial surveys and health checkups for all participants.^22^ The data collection procedure and variable definitions are detailed in eMethods in the Supplement, as well as previous publications from the Kailuan study.^34,35^

### Statistical Analysis

Because statin treatment was not randomized, substantial differences in baseline characteristics existed between participants who took statins and those who did not; therefore, a propensity score–matched analysis was applied. We included as many variables as possible in the propensity score model to reduce treatment selection bias (details are shown in eMethods in the Supplement). Then, we calculated a propensity score for each patient, and patients were matched 1-to-1 without replacement using a nearest neighbor approach with a caliper width of 0.20 SD.^36^ Standardized mean biases were evaluated to ensure balance after propensity score matching between the statin and non–statin groups, and a between-group difference of less than 0.10 was considered to be well balanced.^37^

Multivariable linear regression models were used to estimate the associations of statin use with baseline baPWV and progression of baPWV. In the analysis of statin use and baseline baPWV, all standardized differences in baseline covariates between the 2 groups were less than 0.1 except for age, MAP, and fasting blood glucose (FBG), which were further adjusted in the linear regression models.^38^ In the analysis of statin use and progression of baPWV, age and follow-up duration were adjusted in the linear regression models together with baseline baPWV. We further explored changes in anthropometric measurements and blood biochemical indicators between the statin and non–statin groups in the matched cohort.

Stratified analyses were conducted by baseline characteristics, including age groups (<60 years and ≥60 years), sex, hs-CRP groups (<0.1 mg/dL and ≥0.1 mg/dL [to convert to milligrams per liter, multiply by 10]), smoking status, alcohol intake, physical activity, salt intake, dyslipidemia, type 2 diabetes, hypertension, metabolic syndrome, obesity, and antihypertensive and antidiabetic drugs use. We also performed several sensitivity analyses to assess the robustness of the results. First, multivariable-adjusted logistic regression models were used to estimate the odds ratios and the corresponding 95% CIs for the associations between statin use and elevated arterial stiffness, which was defined as baseline baPWV of 1800 cm/s or higher.^39^ Second, we performed analyses in the overall study participants without propensity-matched analysis (n = 5105 for baseline baPWV analysis and n = 1502 for progression of baPWV). Third, given that behaviors and other covariates might be changed during follow-up, we further adjusted follow-up covariates for analysis in the matched participants. Fourth, statin discontinuation was defined as the absence of statin prescriptions for 3 or 6 consecutive months within the first 2 years of outcome measurements.^40,41^ Fifth, we included those who had CVD during follow-up (n = 49) in the analysis of progression of arterial stiffness.

All analyses were conducted with SAS version 9.3 (SAS Institute), and a 2-sided *P* < .05 was considered as statistical significance. Data were analyzed from February 2021 to April 2022.

## Results

### Participants’ Characteristics

Table 1 shows the baseline characteristics of 5105 participants with baPWV assessment (mean [SD] age: 60.8 [9.7] years; 3842 [75.3%] men and 1263 [24.7%] women) before and after propensity score matching. Participants in the statin group, compared with participants in the non–statin group, were older (mean [SD] age, 64.2 [8.8] years vs 59.3 [9.8] years), had higher prevalence of hypertension (1480 participants [94.2%] vs 2556 participants [72.3%]) and type 2 diabetes (723 participants [46.0%] vs 1433 participants [40.6%]), and higher proportion of antihypertensive drug use (1395 participants [88.8%] vs 1135 participants [32.1%]) and antidiabetic drug use (634 participants [40.4%] vs 1014 participants [28.7%]) before propensity score matching. After matching, 2620 participants (1310 in the statin group and 1310 in the non–statin group) were included in the final analysis (mean [SD] age, 63.2 [9.3] years; the baseline characteristics of the statin group and non–statin group were well balanced (Table 1), except for age, MAP, and FBG. A comparison of participants with and without baPWV assessments revealed that participants who received baPWV measurements were generally older, had higher body mass index (BMI, calculated as weight in kilograms divided by height in meters squared), MAP, FBG, triglycerides, LDL-C, heart rate, and hs-CRP, and they were more likely to have poorer health (standardized differences, >0.10) (eTable 1 in the Supplement).

**Table 1.** Baseline Characteristics of the Participants Before and After Propensity Score Matching

Variables	Participants, No (%)					
	Before matching			After matching		
	Statin group (N = 1571)	Non–statin group (N = 3534)	Standardized mean difference	Statin group (N = 1310)	Non–statin group (N = 1310)	Standardized mean difference
Age, mean (SD), y	64.2 (8.8)	59.3 (9.8)	0.53	64.6 (8.9)	61.9 (9.5)	0.29
Men	1182 (75.2)	2660 (75.3)	−0.002	992 (75.7)	989 (75.5)	0.005
Women	389 (24.8)	874 (24.7)	0.002	318 (24.3)	321 (24.5)	−0.005
BMI, mean (SD)	26.5 (3.2)	26.3 (3.4)	0.07	26.5 (3.2)	26.6 (3.4)	−0.03
MAP, mean (SD), mm Hg	105.2 (13.2)	104.8 (14.0)	0.03	104.8 (13.3)	107.4 (14.5)	−0.19
FBG, mean (SD), mg/dL	117.1 (45.0)	124.3 (52.3)	−0.15	117.1 (46.8)	126.1 (52.3)	−0.19
Triglyceride, mean (SD), mg/dL	194.7 (141.6)	185.8 (159.3)	0.02	194.7 (150.4)	194.7 (168.1)	−0.02
LDL-C, mean (SD), mg/dL	92.7 (50.2)	96.5 (38.6)	−0.09	92.7 (42.5)	96.5 (42.5)	−0.08
HDL-C, mean (SD), mg/dL	57.9 (15.4)	54.1 (15.4)	0.02	57.1 (15.4)	56.4 (15.4)	0.05
Heart rate, mean (SD), bpm	73.9 (11.1)	74.9 (10.6)	−0.09	73.7 (11.1)	74.8 (10.5)	−0.10
Elevated hs-CRP (≥0.1 mg/dL)	1019 (64.9)	2159 (61.1)	0.08	845 (64.5)	852 (65.0)	−0.01
High school or higher	420 (26.7)	929 (26.3)	0.01	343 (26.2)	381 (29.1)	−0.07
Light salt^a^	166 (10.6)	389 (11.0)	−0.01	135 (10.3)	148 (11.3)	−0.03
Current smoker	551 (35.1)	1244 (35.2)	−0.003	465 (35.5)	438 (33.4)	0.04
Alcohol drinking	246 (15.7)	593 (16.8)	−0.03	198 (15.1)	219 (16.7)	−0.04
Physically active^b^	316 (20.1)	607 (17.2)	0.08	265 (20.2)	250 (19.1)	0.03
Atherosclerotic risk factors						
Hypertension	1480 (94.2)	2556 (72.3)	0.61	1219 (93.1)	1215 (92.8)	0.01
Diabetes	723 (46.0)	1433 (40.6)	0.11	605 (46.2)	652 (49.8)	−0.07
Dyslipidemia	790 (50.3)	1758 (49.8)	0.01	659 (50.3)	654 (49.9)	0.01
Metabolic syndrome	691 (44.0)	1400 (39.6)	0.09	558 (42.6)	623 (47.6)	−0.10
Obesity	498 (31.7)	1049 (29.7)	0.04	415 (31.7)	413 (31.5)	0.003
Concurrent drug treatment						
Antihypertensive drug	1395 (88.8)	1135 (32.1)	1.42	1134 (86.6)	1132 (86.4)	0.004
Antidiabetic drug	634 (40.4)	1014 (28.7)	0.25	534 (40.8)	580 (44.3)	−0.07

Abbreviations: BMI, body mass index (calculated as weight in kilograms divided by height in meters squared); bpm, beats per minute; FBG, fasting blood glucose; HDL-C, high density lipoprotein cholesterol; hs-CRP, high-sensitivity C-reactive protein; LDL-C, low-density lipoprotein cholesterol; MAP, mean arterial pressure.

SI conversion factor: to convert FBG to millimoles per liter, multiply by 0.0555; triglyceride to millimoles per liter, multiply by 0.0113; hs-CRP to milligrams per liter, multiply by 10; LDL-C and HDL-C to millimoles per liter, multiply by 0.0259.

^a^ A light salt was defined as less than 6 g/d according to the standard salt spoon in China.

^b^ Being physically active was defined as moderate or vigorous physical activity for at least 80 minutes per week.

Among 5105 participants who received baPWV measurements, 1502 participants (mean [SD] age: 60.5 [9.9] years) received repeated baPWV measurements. Compared with participants in the non–statin group, participants in the statin group were older (63.7 [9.1] years vs 58.9 [9.9] years), had lower LDL-C (88.8 [38.6] mg/dL vs 92.7 [34.7] mg/dL), higher prevalence of hypertension (473 participants [94.0%] vs 764 participants [76.5%]) and type 2 diabetes (337 participants [67.0%] vs 554 participants [55.5%]), and a higher proportion of medication use before propensity score matching. After matching, the characteristics between the 2 groups were well balanced, with all standardized differences less than 0.10 except for age and follow-up duration (eTable 2 in the Supplement). With respect to most of the parameters assessed, the standardized differences between participants with and without repeated baPWV measurements were small (standardized differences, <0.10) (eTable 3 in the Supplement).

### Association Between Statin Use and Arterial Stiffness at Baseline

In the matched cohort, statin use was significantly associated with lower baPWV compared with nonusers (difference, −33.6 cm/s; 95% CI, −62.1 to −5.1 cm/s) (Table 2). A significantly lower baPWV was observed in continuous statin users (difference, −38.6 cm/s; 95% CI, −68.8 to −8.5 cm/s) and high-adherent users (difference, −84.7 cm/s; 95% CI, −124.3 to −45.0 cm/s), but not in discontinued users (difference, −12.9 cm/s; 95% CI, −61.9 to 36.0 cm/s) and low-adherent users (difference, −7.7 cm/s; 95% CI, −39.4 to 24.0 cm/s), compared with non–statin users; a significantly lower baPWV was found in hydrophilic statins users compared with lipophilic statins users (difference, −59.1 cm/s; 95% CI, −105.4 to −12.8 cm/s) (Table 2).

**Table 2.** Associations of Statin Use With baPWV and Progression of baPWV

Variables	Baseline baPWV (N = 2620)		Progression of baPWV (N = 820)	
	Participants, No.	Difference (95% CI)^a^	Participants, No.	Difference (95% CI)^b^
All population				
Statin users	1310	−33.6 (−62.1 to −5.1)	410	−23.3 (−40.6 to −6.0)
Non–statin users	1310	[Reference]	410	[Reference]
Statin discontinuation^c^				
Continuation	1050	−38.6 (−68.8 to −8.5)	354	−24.2 (−42.2 to −6.3)
Discontinuation	260	−12.9 (−61.9 to 36.0)	56	−17.3 (−52.4 to 17.8)
Non–statin users	1310	[Reference]	410	[Reference]
Statin adherence^d^				
High adherence	448	−84.7 (−124.3 to −45.0)	103	−39.7 (−66.9 to −12.4)
Low adherence	862	−7.7 (−39.4 to 24.0)	307	−17.9 (−36.5 to 0.7)
Non–statin users	1310	[Reference]	410	[Reference]
Statin type^e^				
Hydrophilic statins	302	−59.1 (−105.4 to −12.8)	84	−17.7 (−46.3 to 10.8)
Lipophilic statins	1008	[Reference]	326	[Reference]

Abbreviation: baPWV, brachial-ankle pulse wave velocity.

^a^ Adjusted for age, mean arterial pressure, and fasting blood glucose.

^b^ Adjusted for age, baseline baPWV, and duration of baPWV measurement.

^c^ Statin discontinuations were identified as the absence of statin prescriptions for 12 consecutive months within the first 2 years of outcome measurements.

^d^ Medication adherence in the statin group was measured by the medication possession ratio (MPR). High adherence was categorized with MPR values of at least 80%, and low adherence was categorized with MPR values less than 80%.

^e^ Statin type was defined as hydrophilic statins (pravastatin and rosuvastatin) and lipophilic statins (atorvastatin, simvastatin, fluvastatin, lovastatin, and pitavastatin); participants were coalesced into the hydrophilic group because few were hydrophilic users only (n = 59 in baseline baPWV analysis and n = 17 in progression of baPWV analysis) or mixed users of hydrophilic and lipophilic statin (n = 243 in baseline baPWV analysis and n = 67 in progression of baPWV analysis).

### Association Between Statin Use and Progression of Arterial Stiffness

During a mean (SD) of 4.8 (2.7) years of follow-up, the baPWV increased from a mean (SD) of 1778.8 (372.9) cm/s to 1831.9 (396.7) cm/s in the statin group, and from 1799.0 (401.8) cm/s to 1870.8 (407.2) cm/s in the non–statin group (eTable 2 in the Supplement). Multivariable linear regression model showed that statin use was significantly associated with a slower progression of baPWV (difference, −23.3 cm/s per year; 95% CI, −40.6 to −6.0 cm/s per year). In stratified analysis, a significantly slower progression of baPWV was observed in continuous statin users (difference, −24.2 cm/s per year; 95% CI, −42.2 to −6.3 cm/s per year) and high-adherent users (difference, −39.7 cm/s per year; 95% CI, −66.9 to −12.4 cm/s per year), but not in discontinued users (difference, −17.3 cm/s per year; 95% CI, −52.4 to 17.8 cm/s per year) and low-adherent users (difference, −17.9 cm/s per year; 95% CI, −36.5 to 0.7 cm/s per year), compared with non–statin users; no significant difference was found in progression of baPWV between users of hydrophilic or lipophilic statins (difference, −17.7 cm/s per year; 95% CI, −46.3 to 10.8 cm/s per year) (Table 2).

Compared with non–statin users, a significantly slower progression of baPWV was observed in statin users who achieved LDL-C goal as less than 131.3 mg/dL at follow-up (difference, −24.7 cm/s per year; 95% CI, −42.6 to −6.9 cm/s per year), but not in those who did not (difference, −12.6 cm/s per year; 95% CI, −50.2 to 24.9 cm/s per year) (eTable 4 in the Supplement). Table 3 shows changes in anthropometric measurements and blood biochemical indicators between the statin and non–statin groups during follow-up. At baseline, all indexes were well balanced between the groups. During a mean (SD) of 4.8 (2.7) years of follow-up, the statin group showed a greater reduction in LDL-C, hs-CRP, and heart rate. The mean between-group difference was −16.22 (95% CI, −26.64 to −5.41) mg/dL for LDL-C, −0.26 (95% CI, −0.36 to −0.16) mg/dL for hs-CRP, and −2.03 (95% CI, −3.53 to −0.54) beats per minute for heart rate. Mean values or concentrations for other indexes were similar between the 2 groups during follow-up.

**Table 3.** Changes in Anthropometric Measurements and Blood Biochemical Indexes Between the Statin and Non–Statin Groups (N = 820)

Variables	Baseline			Follow-up			Change from baseline^a^	
	Statin group	Non–statin group	*P* value	Statin group	Non–statin group	*P* value	Difference (95% CI)	*P* value
MAP, mean (SD), mm Hg	104.6 (13.5)	105.3 (14.1)	.48	104.5 (12.6)	106.6 (12.9)	.02	−1.60 (−3.19 to −0.01)	.05
BMI, mean (SD), kg/m^2^	26.5 (3.2)	26.5 (3.3)	.99	26.4 (3.2)	26.6 (3.3)	.39	−0.16 (−0.54 to 0.22)	.40
Heart rate, mean (SD), bpm	74.5 (11.4)	74.1 (10.2)	.59	76.4 (11.3)	78.3 (12.6)	.03	−2.03 (−3.53 to −0.54)	.008
FBG, mean (SD), mg/dL	122.5 (50.4)	124.3 (46.8)	.58	144.1 (59.5)	142.3 (57.7)	.58	3.96 (−2.70 to 10.63)	.24
hs-CRP, mean (SD), mg/dL	0.3 (0.5)	0.3 (0.9)	.73	0.1 (0.3)	0.4 (1.0)	<.001	−0.26 (−0.36 to −0.16)	<.001
Triglyceride, mean (SD), mg/dL	203.5 (150.4)	203.5 (168.1)	.98	212.4 (185.8)	203.5 (212.4)	.55	9.73 (−14.16 to 34.51)	.42
LDL-C, mean (SD), mg/dL	92.7 (38.6)	92.7 (34.7)	.59	108.1 (38.6)	123.6 (100.4)	.005	−16.22 (−26.64 to −5.41)	.003
HDL-C, mean (SD), mg/dL	57.9 (15.4)	56.0 (15.4)	.22	57.9 (19.3)	61.8 (57.9)	.26	−3.86 (−10.04 to 1.93)	.19

Abbreviations: baPWV, brachial-ankle pulse wave velocity; BMI, body mass index (calculated as weight in kilograms divided by height in meters squared); bpm, beats per minute; FBG, fasting blood glucose; HDL-C, high density lipoprotein cholesterol; hs-CRP, high-sensitivity C-reactive protein; LDL-C, low-density lipoprotein cholesterol; MAP, mean arterial pressure.

SI conversion factor: to convert FBG to millimoles per liter, multiply by 0.0555; triglyceride to millimoles per liter, multiply by 0.0113; LDL-C and HDL-C to millimoles per liter, multiply by 0.0259; and hs-CRP to milligrams per liter, multiply by 10.

^a^ Mean changes from baseline were estimated from multivariable linear regression models by adjusting for age and duration of baPWV. Baseline values of each index were separately adjusted in the model.

### Subgroup and Sensitivity Analyses

**Figure 2.**
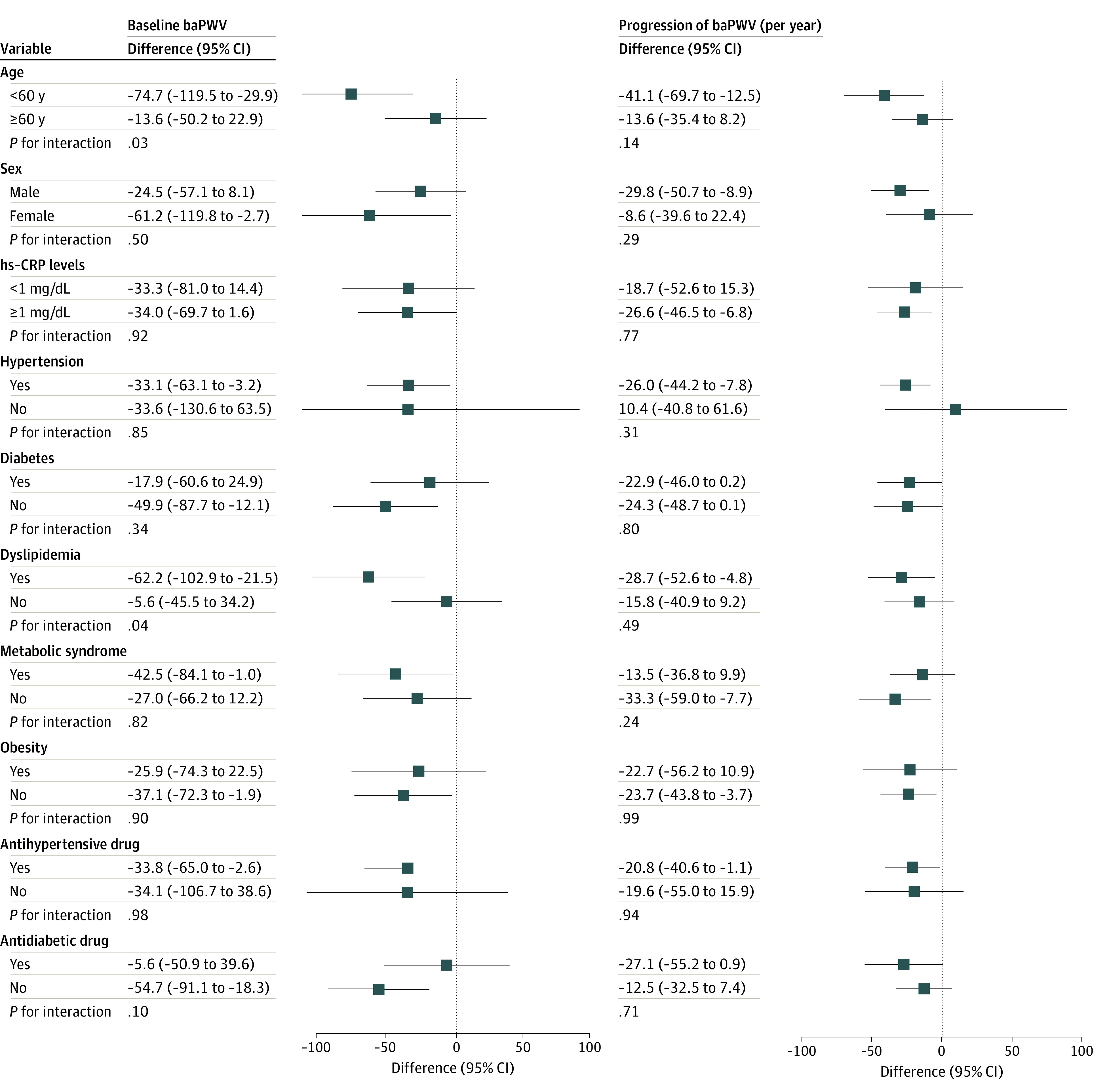
Subgroup Analyses for the Associations of Statin Use With Brachial-Ankle Pulse Wave Velocity (baPWV) and Its Progression According to Baseline Characteristics hs-CRP indicates high-sensitivity C-reactive protein. To convert hs-CRP to milligrams per liter, multiply by 10.

The associations between statin use and baPWV were consistent in the prespecified subgroups except for baseline age (*P* value of interaction = .03) and dyslipidemia (*P* value of interaction = .04) (Figure 2 and the eFigure in the Supplement). A significantly lower baseline baPWV was observed among those younger than 60 years (−74.7 cm/s [95% CI, −119.5 to −29.9 cm/s]) and those with dyslipidemia (−62.2 cm/s [95% CI, −102.9 to −21.5 cm/s]), but not in those aged 60 years or older (−13.6 cm/s [95% CI, −50.2 to 22.9 cm/s]) and those without dyslipidemia (−5.6 cm/s [95% CI, −45.5 to 34.2 cm/s]). Similar results were found in sensitivity analyses for statin use and elevated arterial stiffness defined by baPWV of 1800 cm/s or more (eTable 5 in the Supplement); using multivariable-adjusted analyses in the overall study participants without propensity-matched analysis (eTable 6 in the Supplement) or in the matched participants (eTable 7 in the Supplement); identifying statin discontinuation as the absence of statin prescriptions for 3 or 6 months within the first 2 years of outcome measurements (eTable 8 in the Supplement); and including those who had CVD during follow-up in the progression of arterial stiffness (eTable 9 in the Supplement).

## Discussion

To our knowledge, this is the largest and longest study assessing the association between statin use and progression of arterial stiffness in adults with high atherosclerotic risk. The present study found that statin use was significantly associated with lower baseline baPWV and slower progressions of baPWV over a mean follow-up of 4.8 years. Moreover, continuous and high-adherence use were associated with greater treatment benefit than discontinuation and low-adherence use.

Prior studies have reported mixed findings regarding the association between statin use and PWV in patients with high atherosclerotic risk. A significant reduction in PWV with statin use was observed in several studies with relatively small sample sizes.^10,11,12,14,15,16,19,20^ However, a French study^13^ found that aortic PWV significantly increased by 8% after receiving 12-week atorvastatin or placebo among patients with hypertension and hypercholesterolemia. In contrast, others did not observe a significant change in PWV among patients with hypercholesterolemia.^17,18^ Possible reasons for this inconsistency include varied intensity of statins across studies, small sample sizes (eg, generally <100),^10,11,12,13,14,15,16,17,18,19,20^ heterogeneous populations, and short period of interventions (eg, generally ≤6 months).^10,11,12,13,19,20^ In addition, most studies only compared PWV values pretreatment and posttreatment and did not include non–statin users,^12,15,16,17,18,20^ and some studies combined statins and diet as the intervention.^12,14^

In the present study, the mean baPWV increased in both statin and non–statin groups during the follow-up, while the non–statin group had greater increases. Recently, within the same cohort, Wu et al^42^ showed that baPWV increased by 10.9 cm/s per year among adults without CVD. The effect of statin use on the decelerated progression of baPWV in the present study (−23.3 cm/s per year) was equivalent to 2 times the age-related progression of arterial stiffness and approached 4 times for high-adherent statin users (−39.7 cm/s per year). Although pharmacological treatment (eg, antihypertensive drugs) and lifestyle modifications have been shown to exert a modulatory effect on arterial stiffness, there is still no consensus on the best protective prescriptions for vascular function.^43^ At present, evidence for statin use and progression of arterial stiffness is sparse. Our findings suggest that statin use might provide a substantial potential in decelerating progression of arterial stiffness and preventing the development of subclinical cardiovascular lesions at an early stage.

The association of statin use on arterial stiffness may be due to their pleiotropic effects, such as anti-inflammatory, anti-proliferative, antioxidant, immunomodulatory, and antithrombotic properties.^44^ For example, we observed a significant reduction in LDL-C (mean, −16.22 mg/dL) and hs-CRP (mean, −0.26 mg/dL) in statin group compared with non–statin group, which was consistent with previous studies.^14,45^ Moreover, statins could reduce endothelial dysfunction, improve vascular remodeling, and stabilize atherosclerotic plaque.^8,46^

Furthermore, recent literature has highlighted the importance of statin adherence. Rodriguez et al^29^ examined 347 104 CVD patients from the US Veterans Affairs Health System and observed that patients with the lowest statin adherence levels (MPR <50%) had a hazard ratio for all-cause mortality of 1.30 (95% CI, 1.27-1.34) as compared with the most adherent patients (MPR ≥90%). Using data from the Danish Health Data Authority, Thompson et al^41^ found that 30% of older people (n = 8311) in Denmark discontinued statin use in the primary prevention cohort. In our study, about one-fifth and two-thirds of statin users had either discontinued use or had low-adherent use, respectively. More aggressive efforts are needed to identify reasons for statin discontinuation and develop management strategies that could sustain statin use among adults with high atherosclerotic risk.

### Limitations

This study had several limitations. First, this was an observational study and statins were not randomized, which might result in systematic differences in baseline characteristics. However, we performed a propensity-based method in our attempt to achieve balanced characteristics between the 2 groups. The findings were consistent in the matched and overall population, indicating the robustness of our results. Second, selection bias might exist as the baPWV assessment was voluntary and some participants did not attend the baPWV measurements at baseline or during the follow-up. Third, we used baPWV as the measurement of arterial stiffness instead of carotid femoral PWV (cfPWV), the criterion standard. However, evidence showed that baPWV was closely correlated with cfPWV and the predictive value of baPWV for clinical outcomes was similar to that of cfPWV.^47^ Therefore, using noninvasive measurement and brachial cuff-based waveform analysis, baPWV has simplified the procedure and provided better reproducibility, making it more applicable in large epidemiological studies.^48^ Thus, the American Heart Association recommended baPWV as a common indicators for arterial stiffness.^1^

Fourth, we did not include prescriptions in other hospitals because of lack of information. We deemed the proportion could be small because the Kailuan Group has its own health care system, including the affiliated hospitals and reimbursement system.^23^ For example, a secondary reimbursement can be applied in addition to the statutory health insurance benefits for patients hospitalized in the affiliated hospitals. Therefore, if the participants wanted to get more reimbursement for their treatment, they needed to go to the affiliated hospitals. In addition, participants who received health checkups in the Kailuan General Hospital preferred to see a doctor in this hospital because it was the best hospital in the Kailuan Group and participants usually lived nearby. Fifth, we did not have information on doses of statin for each prescription and further studies including doses of statin are needed to explore the dose-response association between statin use and arterial stiffness. Sixth, the study population was limited to adults with high atherosclerotic risk from the Kailuan study, and our estimates might not be readily generalizable to individuals from other races and regions because of marked differences in clinical and metabolic characteristics among different populations.

## Conclusions

This retrospective cohort study found that statin use is associated with slowing the progression of baPWV among adults with high atherosclerotic risk, especially among patients who continuously take statins and have high adherence. These findings suggest that statin use provides a substantial potential in preventing the development and worsening of subclinical cardiovascular lesions at an early stage. Prospective studies are needed to validate this association.

## Notes

Supplement.
eMethods.
**eTable 1. **Baseline Characteristics for Participants Who Received the baPWV Measurement and Those Who Did Not **eTable 2. **Baseline Characteristics of the Participants Before and After Propensity Score-Matching for Progression of baPWV Analysis **eTable 3. **Baseline Characteristics for Participants Who Received Repeated baPWV Measurement and Those Who Did Not **eTable 4. **Associations of Statin Use With Progression of baPWV: Stratified by the Achieved Lipid Goals **eTable 5. **Sensitivity Analysis for Statin Use and Arterial Stiffness Status Using ≥1800 as Cutoff **eTable 6. **Sensitivity Analysis for Statin Use and Progression of Arterial Stiffness in the Overall Study Participants Without Propensity-Matched Analysis **eTable 7. **Sensitivity Analysis for Statin Use and Progression of Arterial Stiffness With Multivariable Adjustment in the Matched Sample **eTable 8.** Sensitivity Analysis for Statin Use With Baseline baPWV and Progression of baPWV by Different Definition of Statin Discontinuation **eTable 9. **Sensitivity Analysis for Statin Use and Progression of Arterial Stiffness by Including Those Who Had CVD During Follow-up in the Progression of Arterial Stiffness **eFigure. **Subgroup Analyses for the Associations of Statin Use With baPWV and Its Progression According to Baseline Characteristics
