# Supplementary material for: Association Between Statin Use and Progression of Arterial Stiffness Among Adults With High Atherosclerotic Risk
Source: JAMA Netw Open. 2022 Jun 17;5(6):e2218323. doi: 10.1001/jamanetworkopen.2022.18323 (PMC9206193; doi:10.1001/jamanetworkopen.2022.18323)
Supplement: Supplement. — eMethods. eTable 1. Baseline Characteristics for Participants Who Received the baPWV Measurement and Those Who Did Not eTable 2. Baseline Characteristics of the Participants Before and After Propensity Score-Matching for Progression of baPWV Analysis eTable 3. Baseline Characteristics for Participants Who Received Repeated baPWV Measurement and Those Who Did Not eTable 4. Associations of Statin Use With Progression of baPWV: Stratified by the Achieved Lipid Goals eTable 5. Sensitivity Analysis for Statin Use and Arterial Stiffness Status Using ≥1800 as Cutoff eTable 6. Sensitivity Analysis for Statin Use and Progression of Arterial Stiffness in the Overall Study Participants Without Propensity-Matched Analysis eTable 7. Sensitivity Analysis for Statin Use and Progression of Arterial Stiffness With Multivariable Adjustment in the Matched Sample eTable 8. Sensitivity Analysis for Statin Use With Baseline baPWV and Progression of baPWV by Different Definition of Statin Discontinuation eTable 9. Sensitivity Analysis for Statin Use and Progression of Arterial Stiffness by Including Those Who Had CVD During Follow-up in the Progression of Arterial Stiffness eFigure. Subgroup Analyses for the Associations of Statin Use With baPWV and Its Progression According to Baseline Characteristics [file jamanetwopen-e2218323-s001.pdf]

Supplemental Online Content

Zhou YF, Wang Y, Wang G, et al. Association between statin use and progression of arterial stiffness among adults with high atherosclerotic risk. *JAMA Netw Open*. 2022;5(6):e2218323. doi:10.1001/jamanetworkopen.2022.18323

eMethods.
eTable 1. Baseline Characteristics for Participants Who Received the baPWV Measurement and Those Who Did Not
eTable 2. Baseline Characteristics of the Participants Before and After Propensity Score-Matching for Progression of baPWV Analysis
eTable 3. Baseline Characteristics for Participants Who Received Repeated baPWV Measurement and Those Who Did Not
eTable 4. Associations of Statin Use With Progression of baPWV: Stratified by the Achieved Lipid Goals
eTable 5. Sensitivity Analysis for Statin Use and Arterial Stiffness Status Using ≥1800 as Cutoff
eTable 6. Sensitivity Analysis for Statin Use and Progression of Arterial Stiffness in the Overall Study Participants Without Propensity-Matched Analysis
eTable 7. Sensitivity Analysis for Statin Use and Progression of Arterial Stiffness With Multivariable Adjustment in the Matched Sample
eTable 8. Sensitivity Analysis for Statin Use With Baseline baPWV and Progression of baPWV by Different Definition of Statin Discontinuation
eTable 9. Sensitivity Analysis for Statin Use and Progression of Arterial Stiffness by Including Those Who Had CVD During Follow-up in the Progression of Arterial Stiffness
eFigure. Subgroup Analyses for the Associations of Statin Use With baPWV and Its Progression According to Baseline Characteristics

This supplemental material has been provided by the authors to give readers additional information about their work.

© 2022 Zhou YF et al. *JAMA Network Open.*

eMethods.

Study participants

According to clinical guidelines, statin therapy was recommended in addition to lifestyle therapy for patients with hypertension, type 2 diabetes, dyslipidemia, obesity, or metabolic syndrome.[1-3] Hypertension was defined as defined self-reported hypertension, current use of antihypertensive medications, or persistently elevated blood pressure (systolic/diastolic blood pressure ≥140/90 mm Hg). Type 2 diabetes was defined as either fasting blood glucose (FBG) ≥7.0 mmol/L, self-report of a physician diagnosis, or self-reported use of antidiabetic medication. Dyslipidemia was defined as either or combination of serum total cholesterol (TC) ≥6.2 mmol/L (240 mg/dL), low-density lipoprotein cholesterol (LDL-C) ≥4.1 mmol/L (160 mg/dL), high-density lipoprotein cholesterol (HDL-C) <1.04 mmol/L (40 mg/dL), triglycerides ≥2.3 mmol/L (200 mg/dL), or self-reported use of lipid-lowering medication.[2] Obesity was defined as a body mass index (BMI) ≥28 kg/m^2 according to the Working Group on Obesity in China.[4] Metabolic health was defined in accordance with the National Cholesterol Education Program Adult Treatment Panel III (NECP-ATP-III) criteria for metabolic syndrome.[3]

Assessment of covariates

The data collection procedure and variable definitions were described in detail in previous publications from the Kailuan study.[5] BMI was calculated as weight (kg) divided by height squared (m^2). Participants who currently smoked were defined as current smokers, and those who currently drank were defined as current drinkers. Physical activity was evaluated from responses to questions regarding the frequency of physical activity (≥20 min/time) during leisure time, and being physical active was defined as moderate or vigorous physical activity for ≥80 minutes per week.[6] Salt intake was assessed by asking participants to rate their habitual daily salt intake as 'low,' 'medium' or 'high' with a definition of <6 g/day, 6-12 g/day, ≥12 g/day, respectively, and salt light was defined as <6 g/day according to the standard salt spoon in China.[6]

All participants underwent blood tests after overnight fasting at each physical examination. The blood samples were analyzed using an auto-analyzer (Hitachi 747, Hitachi, Tokyo, Japan) at the central laboratory of Kailuan hospital.[7] Fasting blood glucose (FBG) was measured using the hexokinase/ glucose-6-phosphate dehydrogenase method (Mind Bioengineering Co Ltd, Shanghai, China) with an upper limit of detection of 30.07 mmol/L.[7]

Statistical analysis

Because statin treatment was not randomized, substantial differences in baseline characteristics existed between participants who took statins and those who did not; therefore, a propensity score-matched analysis was applied. We included as many variables as possible in the propensity score model to reduce treatment selection bias, including age, sex, educational level (junior high school and below, senior high school and above), body mass index (BMI), MAP, fasting blood glucose (FBG), low-density lipoprotein cholesterol (LDL-C), high-density lipoprotein cholesterol (HDL-C), triglycerides, heart rate, hs-CRP (<0.1 and ≥0.1 mg/dL),[7] smoking habits (current smoker and non-current smoker), alcohol use (current drinker and non-current drinker), physical activity (physically active and physically inactive),[6] salt intake (self-reported light and non-light salt intake),[5] medications use (antidiabetic and antihypertensive drugs), atherosclerotic risk factors (dyslipidemia, type 2 diabetes, hypertension, metabolic syndrome, and obesity), and follow-up duration (for progression of baPWV only). Then, we calculated a propensity score for each subject, and subjects were matched one-to-one without replacement using a nearest-neighbour approach with a caliper width of 0.20 standard deviations (SD).[8] Standardized mean biases were evaluated to ensure balance after propensity score matching between the statin and non-statin groups, and a between-group difference of less than 0.10 was considered to be well balanced.[9]

References

1. American Diabetes Association. 10. Cardiovascular Disease and Risk Management: Standards of Medical Care in Diabetes-2021. *Diabetes Care*. 2021;44:S125-S150. doi: 10.2337/dc21-S010
2. Joint Committee for Developing Chinese guidelines on Prevention and Treatment of Dyslipidemia in Adults. Chinese guidelines on preventin and treatment of dyslipidemia in adults [in Chinese]. *Zhonghua Xin Xue Guan Bing Za Zhi*. 2007;35:390-419.
3. Alberti KG, Eckel RH, Grundy SM, et al. Harmonizing the metabolic syndrome: a joint interim statement of the International Diabetes Federation Task Force on Epidemiology and Prevention; National Heart, Lung, and Blood Institute; American Heart Association; World

© 2022 Zhou YF et al. *JAMA Network Open.*

Heart Federation; International Atherosclerosis Society; and International Association for the Study of Obesity. *Circulation*. 2009;120:1640-5. doi: 10.1161/CIRCULATIONAHA.109.192644
4. Pan XF, Wang L and Pan A. Epidemiology and determinants of obesity in China. *Lancet Diabetes Endocrinol*. 2021;9:373-392. doi: 10.1016/S2213-8587(21)00045-0
5. Li Y, Huang Z, Jin C, et al. Longitudinal Change of Perceived Salt Intake and Stroke Risk in a Chinese Population. Stroke. 2018;49:1332-1339. doi: 10.1161/STROKEAHA.117.020277
6. Wu S, Huang Z, Yang X, et al. Prevalence of ideal cardiovascular health and its relationship with the 4-year cardiovascular events in a northern Chinese industrial city. Circ Cardiovasc Qual Outcomes. 2012;5:487-93. doi: 10.1161/CIRCOUTCOMES.111.963694
7. Wu Z, Huang Z, Jin W, et al. Peripheral Inflammatory Biomarkers for Myocardial Infarction Risk: A Prospective Community-Based Study. Clin Chem. 2017;63:663-672. doi: 10.1373/clinchem.2016.260828
8. Seeger JD, Williams PL, Walker AM. An application of propensity score matching using claims data. Pharmacoepidemiology and drug safety. 2005;14(7):465-476. doi: 10.1002/pds.1062
9. Austin PC. Balance diagnostics for comparing the distribution of baseline covariates between treatment groups in propensity-score matched samples. Stat Med. 2009;28(25):3083-3107. doi: 10.1002/sim.3697

© 2022 Zhou YF et al. *JAMA Network Open.*

eTable 1. Baseline Characteristics for Participants Who Received the baPWV Measurement and Those Who Did Not

Variables	Received baPWV measurement (N=5242)	Did not receive baPWV measurement (N=8263)	Standardized differences
Mean (SD) age (years)	60.8 (9.7)	51.3 (12.5)	0.85
Men	3948 (75.3)	6249 (75.6)	-0.007
Mean (SD) MAP (mm Hg)	104.9 (13.8)	99.7 (12.5)	0.39
Mean (SD) BMI (kg/m^2)	26.4 (3.4)	26.0 (3.4)	0.12
Mean (SD) FBG (mg/dL)	120.7 (50.4)	97.3 (28.8)	0.57
Mean (SD) triglyceride (mg/dL)	194.7 (159.3)	168.1 (123.9)	0.19
Mean (SD) LDL-C (mg/dL)	96.5 (42.5)	88.8 (30.9)	0.21
Mean (SD) HDL-C (mg/dL)	57.9 (15.4)	57.9 (15.4)	<0.001
Mean (SD) heart rate (bpm)	74.6 (10.7)	73.4 (10.9)	0.11
Elevated hs-CRP (≥0.1 mg/dL)	3265 (62.3)	4218 (51.1)	0.23
High school or higher	1385 (26.4)	2983 (36.1)	-0.21
Light salt [a]	574 (11.0)	1000 (12.7)	-0.05
Current smoker	1839 (35.1)	3501 (42.4)	-0.15
Alcohol drinking	864 (16.5)	1970 (23.8)	-0.18
Physically active [b]	948 (18.1)	1689 (20.4)	-0.06
Comorbidities			
Hypertension	4101 (78.2)	4221 (51.1)	0.59
Diabetes	2327 (44.4)	795 (9.6)	0.85
Dyslipidemia	2622 (50.0)	4207 (50.9)	-0.02
Metabolic syndrome	2148 (41.0)	2368 (28.7)	0.26
Obesity	1614 (30.8)	2266 (27.4)	0.07
Concurrent drug treatment			
Antihypertensive drugs	2598 (49.6)	2731 (33.1)	0.34
Antidiabetic drugs	1692 (32.3)	575 (7.0)	0.67

baPWV=brachial-ankle pulse wave velocity; BMI=body mass index; CVD=cardiovascular disease; FBG=fasting blood glucose; MAP=mean arterial pressure; LDL-C=low-density lipoprotein cholesterol; HDL-C=high density lipoprotein cholesterol; hs-CRP=high-sensitivity C-reactive protein.
[a] A light salt was defined as <6 g/day according to the standard salt spoon in China.
[b] Being physically active was defined as moderate or vigorous physical activity for ≥80 minutes per week.
SI conversion factor: To convert FBG to millimoles per liter, multiply by 0.0555; triglyceride to millimoles per liter, multiply by 0.0113; LDL-C and HDL-C to millimoles per liter, multiply by 0.0259.

© 2022 Zhou YF et al. *JAMA Network Open.*

eTable 2. Baseline Characteristics of the Participants Before and After Propensity Score-Matching for Progression of baPWV Analysis

**Variables**	**Before matching (n=1502)**		**Standardized Mean Difference**	**After matching (n=820)**		**Standardized Mean Difference**
	**Statin group (n=503)**	**Non-statin group (n=999)**		**Statin group (n=410)**	**Non-statin group (n=410)**	
Mean (SD) baseline age (years)	63.7 (9.1)	58.9 (9.9)	0.50	63.4 (9.1)	62.3 (9.6)	0.12
Mean (SD) baseline baPWV (cm/s)	1786.5 (373.7)	1727.7 (377.3)	0.17	1778.8 (372.9)	1799.0 (401.8)	-0.05
Mean (SD) duration (years)	5.2 (2.8)	4.1 (2.3)	0.40	5.0 (2.8)	4.5 (2.5)	0.17
Men	333 (66.2)	684 (68.5)	-0.05	269 (65.6)	280 (68.3)	-0.06
Mean (SD) BMI (kg/m^2)	26.7 (3.3)	26.4 (3.4)	0.12	26.5 (3.2)	26.5 (3.3)	<0.001
Mean (SD) MAP (mm Hg)	104.3 (13.5)	103.6 (13.7)	0.06	104.6 (13.5)	105.3 (14.1)	-0.05
Mean (SD) FBG (mg/dL)	118.9 (48.6)	122.5 (50.4)	-0.08	122.5 (50.4)	124.3 (46.8)	-0.04
Mean (SD) triglyceride (mg/dL)	203.5 (150.4)	203.5 (177.0)	-0.01	203.5 (150.4)	203.5 (168.1)	-0.002
Mean (SD) LDL-C (mg/dL)	88.8 (38.6)	92.7 (34.7)	-0.13	92.7 (38.6)	92.7 (34.7)	-0.04
Mean (SD) HDL-C (mg/dL)	57.9 (15.4)	57.9 (15.4)	0.03	57.9 (15.4)	56.0 (15.4)	0.08
Mean (SD) heart rate (bpm)	73.8 (11.0)	74.6 (10.3)	-0.07	74.5 (11.4)	74.1 (10.2)	0.04
Elevated hs-CRP (≥0.1 mg/dL)	336 (66.8)	624 (62.5)	0.09	278 (67.8)	277 (67.6)	0.01
High school or higher	177 (35.2)	372 (37.2)	-0.04	144 (35.1)	164 (40.0)	-0.10
Light salt [a]	57 (11.3)	110 (11.0)	0.01	46 (11.2)	50 (12.2)	-0.03
Current smoker	172 (34.2)	339 (34.0)	0.01	138 (33.7)	134 (32.7)	0.02
Alcohol drinking	77 (15.3)	174 (17.4)	-0.06	61 (14.9)	63 (15.4)	-0.01
Physically active [b]	101 (20.1)	162 (16.2)	0.10	88 (21.5)	74 (18.1)	0.09
Comorbidities						
Hypertension	473 (94.0)	764 (76.5)	0.51	380 (92.7)	378 (92.2)	0.01
Diabetes	337 (67.0)	554 (55.5)	0.24	270 (65.9)	268 (65.4)	0.01
Dyslipidemia	267 (53.1)	499 (50.0)	0.06	216 (52.7)	216 (52.7)	0.00
Metabolic syndrome	243 (48.3)	439 (44.0)	0.09	198 (48.3)	215 (52.4)	-0.07
Obesity	154 (30.6)	296 (29.6)	0.02	124 (30.2)	130 (31.7)	-0.03
Concurrent drug treatment						
Antihypertensive drug	432 (85.9)	346 (34.6)	1.23	339 (82.7)	339 (82.7)	0.00
Antidiabetic drug	276 (54.9)	334 (33.4)	0.44	221 (53.9)	208 (25.4)	0.06

© 2022 Zhou YF et al. *JAMA Network Open.*

baPWV=brachial-ankle pulse wave velocity; BMI=body mass index; CVD=cardiovascular disease; FBG=fasting blood glucose; MAP=mean arterial pressure; LDL-C=low-density lipoprotein cholesterol; HDL-C=high density lipoprotein cholesterol; hs-CRP=high-sensitivity C-reactive protein.
[a] A light salt was defined as <6 g/day according to the standard salt spoon in China.
[b] Being physically active was defined as moderate or vigorous physical activity for ≥80 minutes per week.
SI conversion factor: To convert FBG to millimoles per liter, multiply by 0.0555; triglyceride to millimoles per liter, multiply by 0.0113; LDL-C and HDL-C to millimoles per liter, multiply by 0.0259.

© 2022 Zhou YF et al. *JAMA Network Open.*

eTable 3. Baseline Characteristics for Participants Who Received Repeated baPWV Measurement and Those Who Did Not

Variables	Received repeated baPWV measurement (N=1619)	Did not receive repeated baPWV measurement (N=3486)	Standardized differences
Mean (SD) age (years)	60.9 (9.9)	60.7 (9.7)	0.02
Men	1101 (68.0)	2741 (78.6)	-0.24
Mean (SD) MAP (mm Hg)	103.8 (13.5)	105.4 (13.8)	-0.12
Mean (SD) BMI (kg/m^2)	26.5 (3.4)	26.4 (3.4)	0.03
Mean (SD) FBG (mg/dL)	120.7 (48.6)	122.5 (52.3)	-0.04
Mean (SD) triglyceride (mg/dL)	203.5 (168.1)	185.8 (150.4)	0.11
Mean (SD) LDL-C (mg/dL)	92.7 (34.7)	96.5 (46.3)	-0.09
Mean (SD) HDL-C (mg/dL)	57.9 (15.4)	57.9 (14.7)	<0.001
Mean (SD) heart rate (bpm)	74.3 (10.9)	74.7 (10.7)	-0.04
Elevated hs-CRP (≥0.1 mg/dL)	1045 (64.6)	2133 (61.2)	0.07
High school or higher	587 (36.3)	762 (21.9)	0.32
Light salt [a]	178 (11.0)	377 (10.8)	0.006
Current smoker	552 (34.1)	1243 (35.7)	-0.03
Alcohol drinking	265 (16.4)	574 (16.5)	-0.003
Physically active [b]	278 (17.2)	645 (18.5)	-0.03
Comorbidities			
Hypertension	1333 (82.3)	2736 (78.5)	0.09
Diabetes	960 (59.3)	1345 (38.6)	0.42
Dyslipidemia	827 (51.1)	1271 (49.4)	0.03
Metabolic syndrome	732 (45.2)	1359 (39.0)	0.12
Obesity	503 (31.1)	1052 (30.2)	0.02
Concurrent drug treatment			
Antihypertensive drugs	885 (54.7)	1645 (47.2)	0.15
Antidiabetic drugs	638 (39.4)	1010 (29.0)	0.22

baPWV=brachial-ankle pulse wave velocity; BMI=body mass index; CVD=cardiovascular disease; FBG=fasting blood glucose; MAP=mean arterial pressure; LDL-C=low-density lipoprotein cholesterol; HDL-C=high density lipoprotein cholesterol; hs-CRP=high-sensitivity C-reactive protein.
[a] A light salt was defined as <6 g/day according to the standard salt spoon in China.
[b] Being physically active was defined as moderate or vigorous physical activity for ≥80 minutes per week.
SI conversion factor: To convert FBG to millimoles per liter, multiply by 0.0555; triglyceride to millimoles per liter, multiply by 0.0113; LDL-C and HDL-C to millimoles per liter, multiply by 0.0259.

© 2022 Zhou YF et al. *JAMA Network Open.*

eTable 4. Associations of Statin Use With Progression of baPWV: Stratified by the Achieved Lipid Goals

**Variables**	**Full population (N=1502)**			**Matched population (N=820)**	
	**N**	**Difference (95% CI)** [a]		**N**	**Difference (95% CI)** [b]
Achieved LDL-C goal					
LDL-C<131.3 mg/dL	450	-18.8 (-35.0 to -2.6)		362	-24.7 (-42.6 to -6.9)
LDL-C ≥131.3 mg/dL	53	-9.3 (-44.8 to 26.1)		48	-12.6 (-50.2 to 24.9)
Non-statin users	999	reference		410	reference

[a] Adjusted for sex, baseline age, educational level, MAP, FBG, LDL-C, HDL-C, triglycerides, heart rate, hs-CRP, physical activity, drinking habits, smoking status, salt intake, antihypertensive drugs, antidiabetic drugs, dyslipidemia, type 2 diabetes, hypertension, metabolic syndrome, and obesity, as well as baseline baPWV and duration of baPWV measurement.
[b] Adjusted for age, baseline baPWV, and duration of baPWV measurement.
SI conversion factor: To convert LDL-C to millimoles per liter, multiply by 0.0259.

© 2022 Zhou YF et al. *JAMA Network Open.*

eTable 5. Sensitivity Analysis for Statin Use and Arterial Stiffness Status Using ≥1800 as Cutoff

Variables	The full population (N=5105)			The matched population (N=2620)	
	N	OR (95% CI) [a]		N	OR (95% CI) [b]
All population					
Statin users	1571	0.85 (0.72 to 0.99)		1310	0.83 (0.70 to 0.98)
Non-statin users	3534	reference		1310	reference
Statin discontinuation [c]					
Continuation	1255	0.82 (0.70 to 0.97)		1050	0.82 (0.69 to 0.98)
Discontinuation	316	0.95 (0.73 to 1.25)		260	0.87 (0.65 to 1.16)
Non-statin users	3534	reference		1310	reference
Statin adherence [d]					
High adherence	547	0.69 (0.56 to 0.86)		448	0.68 (0.54 to 0.86)
Low adherence	1024	0.94 (0.79 to 1.11)		862	0.92 (0.76 to 1.11)
Non-statin users	3534	reference		1310	reference
Statin type [e]					
Hydrophilic statins	370	0.80 (0.62 to 1.04)		302	0.76 (0.57 to 1.01)
Lipophilic statins	1201	reference		1008	reference

baPWV, brachial-ankle pulse wave velocity; CI, confidence interval; OR, odds ratio.

[a] Adjusted for age, sex, and educational level, MAP, FBG, LDL-C, HDL-C, triglycerides, heart rate, hs-CRP, physical activity, drinking habits, smoking status, salt intake, antihypertensive, antidiabetic drugs, dyslipidemia, type 2 diabetes, hypertension, metabolic syndrome, and obesity.

[b] Adjusted for age, MAP, and FBG.

[c] Statin discontinuations were identified as the absence of statin prescriptions for 12 consecutive months within the first 2 years of outcome measurements.

[d] Medication adherence in the statin group was measured by the medication possession ratio (MPR). High adherence was categorized with MPR values ≥80% and low adherence was categorized with MPR values <80%.

[e] Statin type was defined as hydrophilic statins (pravastatin and rosuvastatin) and lipophilic statins (atorvastatin, simvastatin, fluvastatin, lovastatin, and pitavastatin); since few participants were hydrophilic users only (n=74 in the full population and n=59 in the matched population) or mixed users of hydrophilic and lipophilic statin (n=296 in the full population and n=243 in the matched population), they were coalesced into hydrophilic group.

eTable 6. Sensitivity Analysis for Statin Use and Progression of Arterial Stiffness in the Overall Study Participants Without Propensity-Matched Analysis

**Variables**	**Baseline baPWV (N=5105)**			**Progression of baPWV (N=1502)**	
	**N**	**OR (95% CI) [a]**		**N**	**OR (95% CI) [b]**
All population					
Statin users	1571	-30.0 (-54.9 to -5.2)		503	-18.2 (-34.0 to -2.5)
Non-statin users	3534	reference		999	reference
Statin discontinuation [c]					
Continuation	1255	-41.2 (-67.5 to -15.0)		435	-19.4 (-35.7 to -3.2)
Discontinuation	316	15.7 (-27.2 to 58.6)		68	-7.3 (-39.5 to 24.8)
Non-statin users	3534	reference		999	reference
Statin adherence [d]					
High adherence	547	-81.6 (-116.3 to -46.8)		127	-25.9 (-50.6 to -1.2)
Low adherence	1024	-3.2 (-31.1 to 24.7)		376	-15.1 (-32.1 to 1.8)
Non-statin users	3534	reference		999	reference
Statin type [e]					
Hydrophilic statins	370	-45.7 (-87.6 to -3.8)		115	-8.8 (-33.6 to 16.0)
Lipophilic statins	1201	reference		388	reference

baPWV, brachial-ankle pulse wave velocity; CI, confidence interval.
[a] Adjusted for sex, baseline age, educational level, MAP, FBG, LDL-C, HDL-C, triglycerides, heart rate, hs-CRP, physical activity, drinking habits, smoking status, salt intake, antihypertensive drugs, antidiabetic drugs, dyslipidemia, type 2 diabetes, hypertension, metabolic syndrome, and obesity.
[b] Adjusted for sex, baseline age, educational level, MAP, FBG, LDL-C, HDL-C, triglycerides, heart rate, hs-CRP, physical activity, drinking habits, smoking status, salt intake, antihypertensive drugs, antidiabetic drugs, dyslipidemia, type 2 diabetes, hypertension, metabolic syndrome, and obesity, as well as baseline baPWV and duration of baPWV measurement.
[c] Statin discontinuations were identified as the absence of statin prescriptions for 12 consecutive months within the first 2 years of outcome measurements.
[d] Medication adherence in the statin group was measured by the medication possession ratio (MPR). High adherence was categorized with MPR values ≥80% and low adherence was categorized with MPR values <80%.
[e] Statin type was defined as hydrophilic statins (pravastatin and rosuvastatin) and lipophilic statins (atorvastatin, simvastatin, fluvastatin, lovastatin, and pitavastatin); since few participants were hydrophilic users only (n=74 in baseline baPWV analysis and n=23 in progression of baPWV analysis) or mixed users of hydrophilic and lipophilic statin (n=296 in baseline baPWV analysis and n=92 in progression of baPWV analysis), they were coalesced into hydrophilic group.

© 2022 Zhou YF et al. *JAMA Network Open.*

eTable 7. Sensitivity Analysis for Statin Use and Progression of Arterial Stiffness With Multivariable Adjustment in the Matched Sample

Variables	Baseline baPWV (N=2620)			Progression of baPWV (N=820)	
	N	Difference (95% CI) [a]		N	Difference (95% CI) [b]
All population					
Statin users	1310	-35.8 (-64.0 to -7.6)		410	-25.2 (-43.0 to -7.5)
Non-statin users	1310	reference		410	reference
Statin discontinuation [c]					
Continuation	1050	-39.1 (-68.9 to -9.2)		354	-25.4 (-43.9 to -7.0)
Discontinuation	260	-22.2 (-70.7 to 26.3)		56	-23.7 (-59.8 to 12.4)
Non-statin users	1310	reference		410	reference
Statin adherence [d]					
High adherence	448	-71.5 (-111.2 to -31.8)		103	-39.9 (-67.9 to -11.9)
Low adherence	862	-17.8 (-49.3 to 13.7)		307	-20.4 (-39.5 to -1.2)
Non-statin users	1310	reference		410	reference
Statin type [e]					
Hydrophilic statins	302	-53.8 (-99.4 to -8.1)		84	-20.5 (-49.9 to 8.9)
Lipophilic statins	1008	reference		326	reference

baPWV, brachial-ankle pulse wave velocity; CI, confidence interval.
[a] Adjusted for sex, baseline age, educational level, MAP, FBG, LDL-C, HDL-C, triglycerides, heart rate, hs-CRP, physical activity, drinking habits, smoking status, salt intake, dyslipidemia, type 2 diabetes, hypertension, metabolic syndrome, obesity, antihypertensive drugs, and antidiabetic drugs.
[b] Adjusted for sex, baseline age, educational level, baseline baPWV, duration of baPWV measurement, and the following variables at baseline and follow-up: MAP, FBG, LDL-C, HDL-C, triglycerides, heart rate, hs-CRP, physical activity, drinking habits, smoking status, salt intake, dyslipidemia, type 2 diabetes, hypertension, metabolic syndrome, obesity, antihypertensive drugs, and antidiabetic drugs.
[c] Statin discontinuations were identified as the absence of statin prescriptions for 12 consecutive months within the first 2 years of outcome measurements.
[d] Medication adherence in the statin group was measured by the medication possession ratio (MPR). High adherence was categorized with MPR values ≥80% and low adherence was categorized with MPR values <80%.
[e] Statin type was defined as hydrophilic statins (pravastatin and rosuvastatin) and lipophilic statins (atorvastatin, simvastatin, fluvastatin, lovastatin, and pitavastatin); since few participants were hydrophilic users only (n=59 in baseline baPWV analysis and n=17 in progression of baPWV analysis) or mixed users of hydrophilic and lipophilic statin (n=243 in baseline baPWV analysis and n=67 in progression of baPWV analysis), they were coalesced into hydrophilic group.

© 2022 Zhou YF et al. *JAMA Network Open.*

eTable 8. Sensitivity Analysis for Statin Use With Baseline baPWV and Progression of baPWV by Different Definition of Statin Discontinuation

Variables	Baseline baPWV (N=2620)			Progression of baPWV (N=820)	
	N	Difference (95% CI) [a]		N	Difference (95% CI) [b]
Statin discontinuation [c]					
Absence of statins for 3 months					
Continuation	698	-38.3 (-69.5 to -7.1)		268	-25.0 (-44.3 to -5.6)
Discontinuation	612	-22.3 (-63.9 to 19.4)		142	-19.7 (-43.7 to 4.3)
Non-statin users	1310	reference		410	reference
Absence of statins for 6 months					
Continuation	917	-35.3 (-69.1 to -1.5)		334	-23.7 (-41.9 to -5.5)
Discontinuation	393	-31.5 (-67.2 to 4.2)		76	-21.4 (-52.3 to 9.4)
Non-statin users	1310	reference		410	reference

baPWV=brachial-ankle pulse wave velocity; CI=confidence interval.
[a] Adjusted for age, MAP, and FBG.
[b] Adjusted for age, baseline baPWV, and duration of baPWV measurement.
[c] Statin discontinuations were identified as the absence of statin prescriptions for 3 or 6 consecutive months within the first 2 years of outcome measurements.

© 2022 Zhou YF et al. *JAMA Network Open.*

eTable 9. Sensitivity Analysis for Statin Use and Progression of Arterial Stiffness by Including Those Who Had CVD During Follow-up in the Progression of Arterial Stiffness

**Variables**	**Progression of baPWV**	
	**N**	**Difference (95% CI)** [a]
All population	1551	
Statin group	543	-16.0 (-31.6 to -0.3)
Non-statin users	1008	reference
Statin discontinuation [b]		
Continuation	463	-19.6 (-38.7 to -0.4)
Discontinuation	80	-8.3 (-28.4 to 11.8)
Non-statin users	1008	reference
Statin adherence [c]		
High adherence	146	-22.4 (-47.6 to 2.8)
Low adherence	397	-11.7 (-29.2 to 5.8)
Non-statin users	1008	reference
Statin type [d]		
Hydrophilic statins	129	-8.4 (-34.9 to 18.0)
Lipophilic statins	414	reference

baPWV, brachial-ankle pulse wave velocity; CI, confidence interval.
[a] Adjusted for sex, baseline age, educational level, occupational type, MAP, FBG, LDL-C, HDL-C, triglycerides, heart rate, hs-CRP, physical activity, drinking habits, smoking status, salt intake, antihypertensive drugs, antidiabetic drugs, dyslipidemia, type 2 diabetes, hypertension, metabolic syndrome, and obesity, as well as baseline baPWV and duration of baPWV measurement.
[b] Statin discontinuations were identified as the absence of statin prescriptions for 12 consecutive months within the first 2 years of outcome measurements.
[c] Medication adherence in the statin group was measured by the medication possession ratio (MPR). High adherence was categorized with MPR values ≥80% and low adherence was categorized with MPR values <80%.
[d] Statin type was defined as hydrophilic statins (pravastatin and rosuvastatin) and lipophilic statins (atorvastatin, simvastatin, fluvastatin, lovastatin, and pitavastatin); since few participants were hydrophilic users only (n=25) or mixed users of hydrophilic and lipophilic statin (n=104), they were coalesced into hydrophilic group.

eFigure. Subgroup Analyses for the Associations of Statin Use With baPWV and Its Progression According to Baseline Characteristics

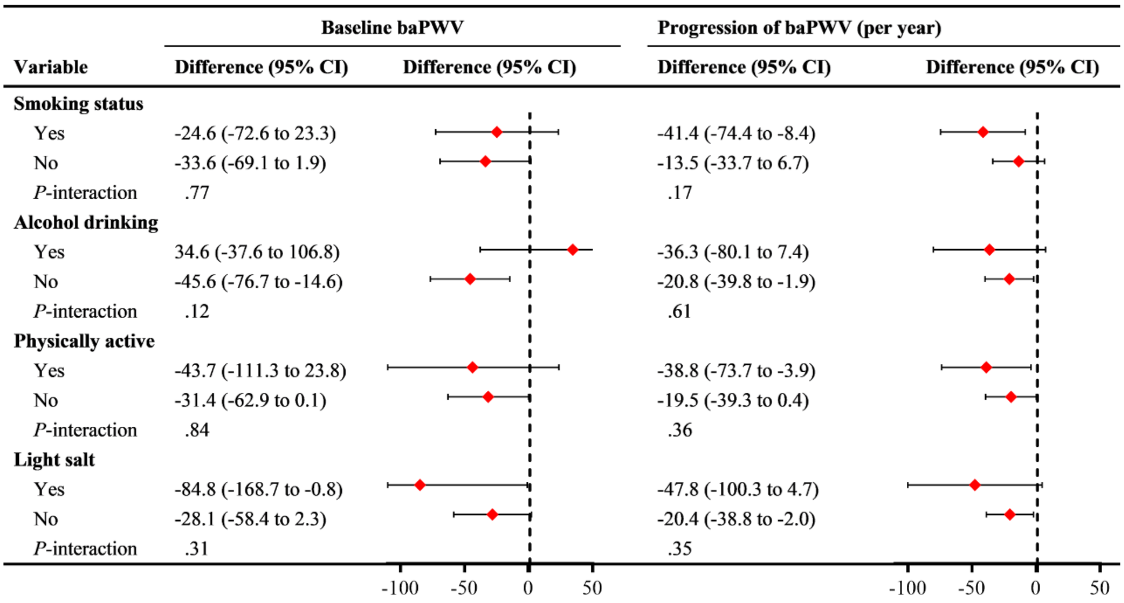

	Baseline baPWV		Progression of baPWV (per year)	
**Variable**	**Difference (95% CI)**	**Difference (95% CI)**	**Difference (95% CI)**	**Difference (95% CI)**
**Smoking status**				
Yes	-24.6 (-72.6 to 23.3)		-41.4 (-74.4 to -8.4)	
No	-33.6 (-69.1 to 1.9)		-13.5 (-33.7 to 6.7)	
P-interaction	.77		.17	
**Alcohol drinking**				
Yes	34.6 (-37.6 to 106.8)		-36.3 (-80.1 to 7.4)	
No	-45.6 (-76.7 to -14.6)		-20.8 (-39.8 to -1.9)	
P-interaction	.12		.61	
**Physically active**				
Yes	-43.7 (-111.3 to 23.8)		-38.8 (-73.7 to -3.9)	
No	-31.4 (-62.9 to 0.1)		-19.5 (-39.3 to 0.4)	
P-interaction	.84		.36	
**Light salt**				
Yes	-84.8 (-168.7 to -0.8)		-47.8 (-100.3 to 4.7)	
No	-28.1 (-58.4 to 2.3)		-20.4 (-38.8 to -2.0)	
P-interaction	.31		.35	

baPWV=brachial-ankle pulse wave velocity; CI=confidence interval.

© 2022 Zhou YF et al. *JAMA Network Open.*
